# Genomic diversity and adaptive resistance mechanisms in *Pseudomonas aeruginosa* from bronchiectasis

**DOI:** 10.1128/msystems.01514-25

**Published:** 2025-12-05

**Authors:** Yanghua Xiao, Jingwen Zhang, Feng Nie, Tingxiu Peng, Ping Li, Keyi Li, Xingyu Tao, Dandan Wei, Fanglin Zheng, Rui Zhao, Wei Zhang

**Affiliations:** 1 Department of Respiratory and Critical Care Medicine, The First Affiliated Hospital, Jiangxi Medical College, Nanchang University 47861, Nanchang, China; 2 Jiangxi Provincial Key Laboratory of Respiratory Diseases, Jiangxi Institute of Respiratory Diseases, The First Affiliated Hospital, Jiangxi Medical College, Nanchang University 47861, Nanchang, China; 3 Department of Clinical Laboratory, Medical Center of Burn plastic and wound repair, The First Affiliated Hospital, Jiangxi Medical College, Nanchang University 47861, Nanchang, China; London School of Hygiene & Tropical Medicine, London, United Kingdom

**Keywords:** *Pseudomonas aeruginosa*, bronchiectasis, genome, adaptation, resistance, virulence

## Abstract

*Pseudomonas aeruginosa* is a predominant colonizer of airways in non-cystic fibrosis bronchiectasis (NCFB), yet its adaptive mechanisms remain poorly understood. This study investigates the genetic characteristics, virulence variation, and resistance mechanisms of 66 *P*. *aeruginosa* isolates derived from NCFB patients. Whole-genome sequencing revealed extensive genetic diversity, encompassing 53 sequence types and a predominance of the O6 serotype (30/66, 45.5%). Phylogenetic analysis indicated that most NCFB isolates were acquired independently, with limited evidence of transmission. Extensive loss-of-function mutations were identified, with *mucA* mutations present in 90.6% (29/32) of mucoid and 67.6% (23/34) of non-mucoid isolates. Most *mucA* mutations were frameshift variants, predominantly at codon 144 (Ala144fs), indicating the selective advantage of this site in driving alginate overproduction during chronic airway infection. Virulence gene profiling demonstrated a highly conserved core repertoire but considerable variability in type VI secretion and pyoverdine systems. Notably, mucoid isolates exhibited significantly higher cefiderocol MICs compared to non-mucoid isolates (*P* = 0.0073), along with enhanced biofilm formation (*P* < 0.0001) but reduced virulence in the *Galleria mellonella* infection model. Mechanistic studies revealed that cefiderocol resistance in mucoid *P. aeruginosa* was driven by synergistic interactions between alginate overproduction and mutations in iron-uptake regulatory genes, particularly Gly132 frameshift in *pirR*. Disruption of alginate biosynthesis (Δ*algD*) and complementation of *pirR* in mucoid strains markedly restored cefiderocol susceptibility. These findings highlight the remarkable genomic diversity and adaptive resistance mechanisms of *P. aeruginosa* in NCFB, providing important insights into its persistence and therapeutic challenges in chronic airway infection.

**IMPORTANCE:**

Understanding the adaptive mechanisms of *Pseudomonas aeruginosa* in non-cystic fibrosis bronchiectasis (NCFB) is critical for improving treatment strategies. This study reveals substantial genomic diversity and highlights alginate overproduction as a key feature of chronic adaptation. Notably, we uncover a novel resistance mechanism involving synergistic interactions between alginate production and mutations in iron-uptake regulators, particularly *pirR*. These findings underscore the complex evolutionary pressures shaping *P. aeruginosa* persistence in NCFB and provide valuable insights into its resistance and virulence balance, offering potential targets for more effective therapeutic interventions.

## INTRODUCTION

*Pseudomonas aeruginosa* is a highly adaptable Gram-negative opportunistic pathogen that frequently colonizes the respiratory tract of patients with chronic lung diseases (1). Compared to other common respiratory pathogens, *P. aeruginosa* is more likely to establish chronic infections and is linked to increased hospitalization rates (2). Non-cystic fibrosis bronchiectasis (NCFB) is a heterogeneous syndrome characterized by irreversible bronchial dilation, chronic airway inflammation, and recurrent infections, ultimately resulting in progressive decline of lung function, impaired quality of life, and increased mortality (3–5). Among the various pathogens implicated in NCFB, *P. aeruginosa* stands out as a major contributor to disease severity, frequent exacerbations, and poor clinical outcomes (2). An increasing body of evidence has highlighted the pivotal role of *P. aeruginosa* in the development and prognosis of NCFB (6–8).

Decades of research in CF have revealed the pathogen’s adaptive evolution, which is characterized by the emergence of mucoid phenotypes, loss of virulence factors, and convergent evolution toward persistence (9–13). However, the population structure, genomic diversity, and adaptive strategies of *P. aeruginosa* in NCFB have only recently begun to be systematically explored. The airway environment in NCFB constitutes a complex ecological niche shaped by unique immunological, anatomical, and microbiological factors, which are distinct from those observed in CF (14). This environment is further characterized by altered mucociliary clearance, epithelial disruption, and persistent inflammation (15). In response to these factors, *P. aeruginosa* may undergo specific genetic and phenotypic adaptations to cope with heterogeneous immune pressures, airway acidification, and diverse etiological challenges (16, 17). This complex interplay between host and microbial determinants is likely to drive the emergence of novel adaptive strategies and resistance mechanisms, many of which remain incompletely characterized and warrant further investigation.

Recent studies have identified that structural lung disease, frequent hospitalizations, and prior antibiotic exposure are risk factors for the emergence of resistant *P. aeruginosa*, with mucoid phenotypes frequently found in patients with recurrent exacerbations and extensive lung involvement (18, 19). The mucoid phenotype, mainly driven by mutations in the *mucA* gene, is closely linked to chronic infection, robust biofilm production, and poor treatment response (18, 20, 21). Although novel agents such as cefiderocol, which targets bacterial iron transport systems (22), exhibit promising therapeutic potential, this drug has not yet been introduced in China, and its effectiveness in NCFB remains to be determined. Moreover, studies have reported that spontaneous mutations in iron transport pathway genes can lead to reduced susceptibility to cefiderocol even in the absence of prior exposure (23). Therefore, elucidating the adaptive strategies and resistance mechanisms of *P. aeruginosa* in NCFB is crucial for optimizing therapeutic strategies and improving clinical management in this patient population.

In this study, we conducted an integrated genomic and phenotypic analysis of 66 *P*. *aeruginosa* isolates obtained from NCFB patients. By characterizing their genomic features, virulence determinants, and antimicrobial resistance, our findings provide new insights into the adaptation strategies of *P. aeruginosa* in chronic NCFB infection.

## MATERIALS AND METHODS

### Isolate collection and identification

A total of 66 non-duplicate clinical *P. aeruginosa* isolates were collected from NCFB patients between 2021 and 2024 at the Department of Respiratory Medicine, First Affiliated Hospital of Nanchang University (Jiangxi, China). All isolates were derived from patients diagnosed with NCFB during their stable phase. The diagnosis of NCFB was established based on recognized criteria (24), including typical clinical symptoms (such as chronic cough, sputum production, or recurrent respiratory infections) and radiological confirmation by high-resolution computed tomography showing columnar or cystic bronchial dilatations. Patients with cystic fibrosis were excluded. Only non-duplicate isolates from patients in the stable phase were included in the study. The stable phase was defined as the absence of acute exacerbation symptoms, including increased cough, sputum purulence or volume, hemoptysis, or dyspnea, for at least 4 weeks prior to sampling, in accordance with established guidelines (25).

### Analysis of mucinous phenotypes of *P. aeruginosa* strains

Mucoid phenotype identification was performed by assessing colony morphology on both Luria-Bertani (LB) and Pseudomonas isolation agar (PIA) plates, as previously described (26). Strains were classified as mucoid if they exhibited typical mucoid transformation on either or both media, according to a four-type system: type I (mucoid on both media), type II (mucoid on PIA only), type III (non-mucoid on both), and type IV (transiently non-mucoid, becoming slightly mucoid after 72 h prolonged incubation). Types I, II, and IV were considered mucoid, while type III was defined as non-mucoid. *P. aeruginosa* PAO1 was used as the quality control strain. All plates were incubated at 37°C for 24–72 h.

### DNA extraction and whole-genome sequencing

Genomic DNA was extracted from overnight cultures of each *P. aeruginosa* isolate using the Sangon Biotech Bacterial Genomic DNA Extraction Kit (Cat. No. B518225-0100), following the manufacturer’s instructions. The quantity and quality of the extracted DNA were assessed using a NanoDrop spectrophotometer (Thermo Scientific, USA) and by agarose gel electrophoresis. Whole-genome sequencing was performed on the DNBSEQ-T7 platform (MGI Tech, Shenzhen, China), utilizing paired-end libraries with a read length of 150 bp. Library preparation was conducted following the manufacturer’s standard protocols.

### Genome assembly and annotation

Raw sequencing reads were first assessed for quality using FastQC v0.11.9 (https://www.bioinformatics.babraham.ac.uk/projects/fastqc/). Adapter sequences and low-quality bases were trimmed using Trimmomatic v0.39 (27). Clean reads were then *de novo* assembled with SPAdes v4.0.0 using default parameters (28). Assembly quality was evaluated using QUAST v5.2.0, and completeness was assessed with BUSCO v5.4.3; any contaminated or poor-quality assemblies were excluded from downstream analysis (29, 30). Genome annotation was performed on the remaining high-quality assemblies with Bakta v1.6.0 (31). All assemblies were also subject to *in silico* sequence typing with mlst v2.11.0, using the PubMLST database (https://pubmlst.org/). The O-antigen serotypes of *P. aeruginosa* isolates were determined *in silico* using the PAst tool (https://github.com/Sandramses/PAst). For any ambiguous results, serotype assignments were further checked by BLASTn analysis of the O-antigen biosynthesis gene clusters. A gene cluster was considered present if it showed ≥80% sequence coverage and ≥90% nucleotide identity compared with the reference. Virulence and antimicrobial resistance genes were identified using ABRicate with the VFDB and ResFinder databases (32, 33), respectively, and were defined as present when they met thresholds of ≥80% sequence coverage and ≥90% nucleotide identity. Genes associated with secretion systems (T1SS, T2SS, T3SS, and T6SS), iron acquisition systems (including the pyochelin and pyoverdine clusters), alginate biosynthesis, and motility genes were selected for analysis, as these represent key determinants of *P. aeruginosa* pathogenicity and environmental adaptability. Accession numbers for all detected genes are listed in Table S1.

### Pangenome and phylogeny construction

Pangenome analysis was performed using Panaroo v1.2.10 to identify core and accessory genes among all *P. aeruginosa* isolates (34). Three reference strains, PAO1 (GCF_000006765.1), PA14 (GCF_000014625.1), and PA7 (GCF_000017205.1), were included to provide phylogenetic context. Core genes were aligned and concatenated, SNPs were extracted with SNP-sites v2.5.1, and pairwise SNP distances were calculated using SNP-dists v0.8.2 (https://github.com/tseemann/snp-dists) (35). A maximum-likelihood phylogenetic tree was constructed from the core SNP alignment using IQ-TREE2 v2.2.6 with ModelFinder and ascertainment bias correction (MFP+ASC, Lewis correction) (36). ModelFinder identified HKY+F+ASC+R7 as the best-fit nucleotide substitution model. Node support was assessed with 1,000 ultrafast bootstrap replicates (UFBoot2) and 1,000 SH-aLRT replicates. The resulting unrooted tree was re-rooted at PA7 as an outgroup and visualized using Interactive Tree of Life (iTOL, https://itol.embl.de) (37).

### Mutation diversity

To investigate mutational diversity among *P. aeruginosa* isolates, SNPs and insertion-deletion mutations (indels) were identified by aligning each assembled genome to the reference genome of *P. aeruginosa* PAO1 (GCF_000006765.1) using Snippy v4.6.0 (https://github.com/tseemann/snippy). The Snippy-core module was subsequently used to generate a core SNP alignment across all isolates, enabling the identification of variants present in conserved genomic regions. Functional annotation and predicted effects of identified variants were performed using SnpEff v5.0 (38). Loss-of-function (LoF) mutations were defined as variants expected to result in complete loss of gene function, including those introducing premature stop codons, disrupting start or stop codons, causing frameshifts or disruptive in-frame insertions/deletions, or leading to gene fusions. To avoid the misclassification of natural strain-specific polymorphisms as loss-of-function mutations, candidate LoF variants were compared against three widely used *P. aeruginosa* reference genomes: PA14 (GCF_000014625.1), PA7 (GCF_000017205.1), and ATCC 27853 (GCF_001687285.1). Any variants detected in these reference genomes were excluded from subsequent analyses of LoF mutations.

### Antimicrobial susceptibility testing

Antimicrobial susceptibility testing was performed using both the disk diffusion method and the broth microdilution method, according to the guidelines of the Clinical and Laboratory Standards Institute (CLSI, 2024). The following antibiotics were tested using the disk diffusion method: ciprofloxacin, aztreonam, cefoperazone/sulbactam, amikacin, tobramycin, and piperacillin/tazobactam. For imipenem, meropenem, cefepime, ceftazidime, ceftazidime-avibactam, colistin, and cefiderocol, minimum inhibitory concentrations (MICs) were determined using the standard broth microdilution method. Cefiderocol susceptibility testing was performed using iron-depleted cation-adjusted Mueller-Hinton broth (ID-CAMHB), as recommended by CLSI. The preparation of ID-CAMHB followed previously described protocols (39), and the final concentration of iron in the prepared ID-CAMHB was ≤0.03  µg/mL. Results were interpreted according to CLSI breakpoints. *Escherichia coli* ATCC 25922 and *P. aeruginosa* ATCC 27853 were used as quality control strains throughout the study.

### Biofilm semi-quantitative assay

Biofilm formation was quantified using the crystal violet staining method as previously described (40). Overnight *P. aeruginosa* cultures were diluted 1:100 in fresh LB medium, and 200 µL aliquots were transferred to 96-well polystyrene microtiter plates in triplicate. Following incubation, planktonic cells were removed by thorough PBS washing. Adherent biofilms were stained with 0.1% crystal violet for 15 min, then washed again with PBS. Bound dye was solubilized using 200 µL of 95% ethanol, and absorbance was measured at 600 nm using a microplate reader. Each isolate was tested in five replicates. After excluding the highest and lowest values, means were calculated from the remaining three measurements. All experiments were repeated three times.

### *Galleria mellonella* larvae infection model

The virulence of *P. aeruginosa* isolates was evaluated *in vivo* using the *G. mellonella* larval infection model. Larvae injected with sterile NaCl were included as uninfected controls. To prepare the inoculum, bacteria were cultured for 12 h at 37°C with shaking at 200 rpm. Bacterial cells were collected by centrifugation at 4,000 × *g*, washed with sterile NaCl, and adjusted to a final concentration of 1 × 10^5^ colony-forming units (CFU)/mL using a Nephelometer (Merieux, Nürtingen, Germany). A 10 µL aliquot (containing 1 × 10^3^ CFU) of each bacterial suspension was injected into the last left proleg of each larva using a Hamilton syringe. After injection, larvae were incubated at 37°C. Larvae were considered dead if they failed to respond to a gentle touch. Survival rates were recorded at 12 h intervals. *P. aeruginosa* PAO1 was included as a high-virulence reference strain, and its mutant PAO1Δ*rhlR* (with the *rhlR* gene deleted) was used as a low-virulence control. Each group contained at least ten larvae, and all experiments were performed in at least three independent biological replicates.

### Construction of *algD* and *rhlR* knockout strains

Gene deletion mutants of *algD* and *rhlR* were generated by allelic exchange, combining λ-Red recombination with a CRISPR/Cas9-based counter-selection system as previously described (41). sgRNA target sequences (20 bp) adjacent to PAM sites within the *algD* and *rhlR* loci were selected, synthesized as complementary oligonucleotides, annealed, and cloned into the pACRISPR vector via Golden Gate assembly. Approximately 500 bp homologous arms flanking the target genes were amplified by PCR and assembled with the pACRISPR-sgRNA construct using Gibson assembly at the XhoI/XbaI sites. All constructs were verified by sequencing in *E. coli* DH5α before being electroporated into PAO1 strains harboring the pCasPA plasmid for arabinose-inducible Cas9 expression. Transformants were selected on LB agar containing 150 µg/mL carbenicillin and 100 µg/mL tetracycline, with Cas9 expression induced by 0.02% L-arabinose. Plasmid curing was achieved by negative selection on LB agar with 5% sucrose. The resulting mutants were confirmed by PCR, agarose gel electrophoresis, and Sanger sequencing. Primer sequences were listed in Table S2.

### Complementation of *algD* and *pirR*

To construct complemented strains, the *algD* and *pirR* genes, together with their native promoter regions, were PCR-amplified from *P. aeruginosa* PAO1 genomic DNA using primer pairs *algD*-C-F/*algD*-C-R and *pirR*-C-F/*pirR*-C-R, respectively (see Table S2). The amplified fragments were seamlessly cloned into the pUCP24 vector via a homologous recombination-based cloning method to generate pUCP24-*algD* and pUCP24-*pirR*. The resulting recombinant plasmids were introduced by electroporation into the relevant recipient strains: the Δ*algD* mutant and the clinical *pirR* frameshift mutant, respectively. Transformants were selected on LB Agar supplemented with 100 µg/mL gentamicin. PCR and Sanger sequencing were performed to confirm the presence and sequence accuracy of the inserted genes in the complemented strains.

### Growth curve analysis

Overnight cultures of *P. aeruginosa* were adjusted to a 0.5 McFarland standard (approximately 1.5 × 10^8^ CFU/mL) and subsequently diluted 1:100 into ID-CAMHB supplemented with cefiderocol at a final concentration of 2 mg/L. An equal volume of solvent (vehicle control) and sterile ID-CAMHB (blank control) were included as controls. All cultures were incubated at 37°C with continuous shaking at 220 rpm in 100-well honeycomb microplates (200 µL per well). Bacterial growth was monitored by measuring the optical density at 600 nm at hourly intervals for 24 h using the Bioscreen C Pro Automated Microbiology Growth Curve Analysis System (Bioscreen C, USA). All experimental conditions were performed in triplicate, and the experiment was independently repeated at least three times to ensure reproducibility.

### Statistical analysis

All statistical analyses were performed using GraphPad Prism 8 (GraphPad Software Inc., San Diego, CA, USA). Differences in MIC values between mucoid and non-mucoid groups were assessed using the unpaired Mann-Whitney test, whereas differences in biofilm-forming ability were analyzed using the unpaired *t*-test. A *P* value <0.05 was considered statistically significant.

## RESULTS

### Epidemiological and genomic diversity of *P. aeruginosa* isolates from NCFB patients

**Fig 1:**
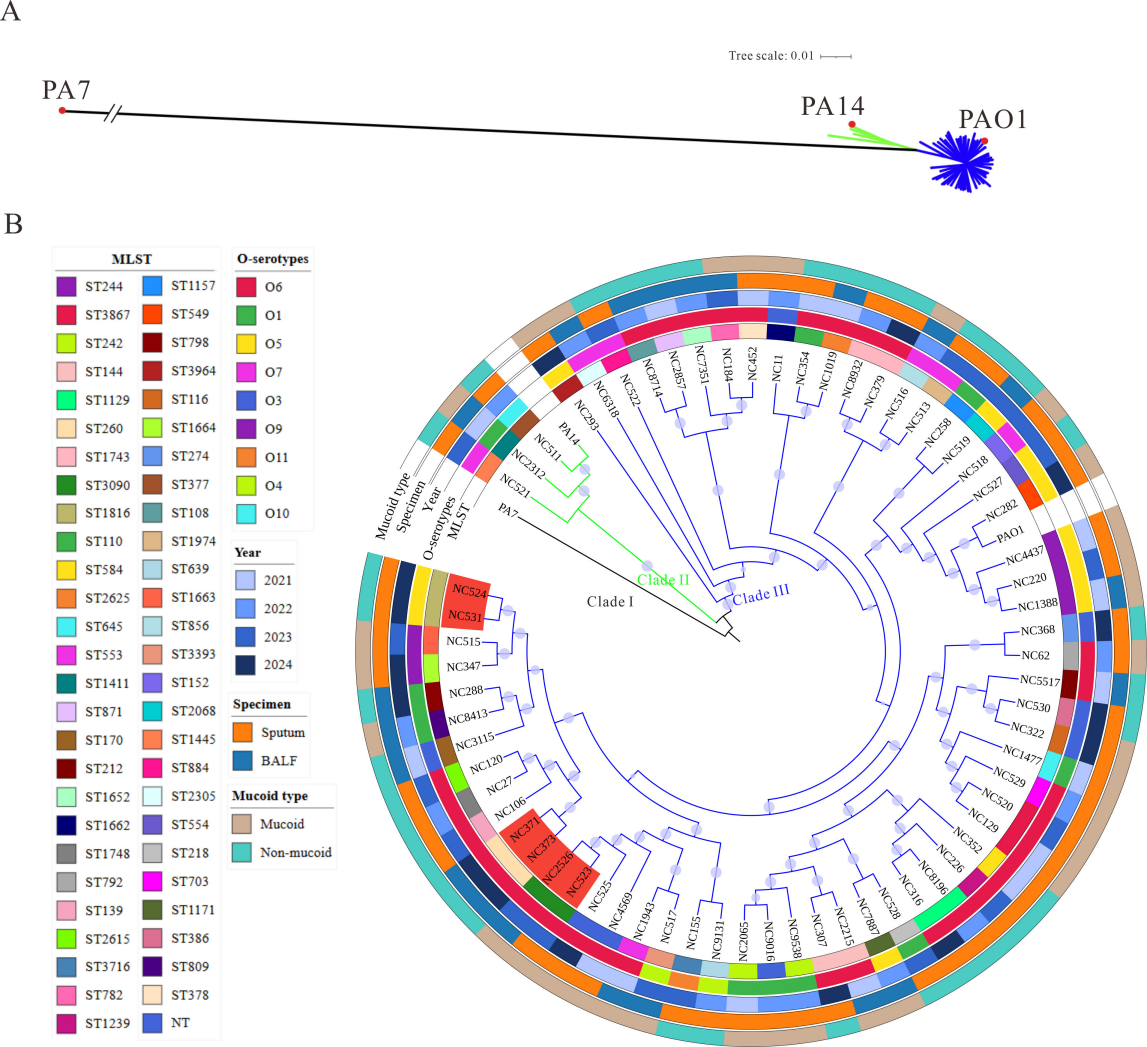
Phylogenetic and molecular characterization of *P. aeruginosa* isolates from NCFB patients. (**A**) Maximum likelihood phylogenetic tree showing the relationship of 66 NCFB isolates to reference strains PAO1, PA14, and PA7, based on the alignment of 4,267 core genes. (**B**) Detailed phylogeny of the 66 NCFB isolates, annotated with multilocus sequence type (MLST), O-serotype, year of isolation, specimen type, and mucoid phenotype.

To elucidate the phylogenetic relationships of NCFB-derived isolates, a maximum likelihood phylogenetic tree was constructed based on the alignment of 4,267 core genes shared across all isolates. As shown in Fig. 1A, the majority of NCFB-derived isolates clustered closely with reference strains PAO1 and PA14. A more detailed phylogenetic analysis of the 66 clinical isolates is presented in Fig. 1B. Most isolates were obtained from sputum samples (48/66, 72.7%), while the remaining were isolated from bronchoalveolar lavage fluid (18/66, 27.3%). Multilocus sequence typing revealed a high degree of genetic heterogeneity, with 53 distinct sequence types (STs) identified among the 66 isolates. ST244 was the most frequently detected (3/66, 4.5%), followed by ST3867, ST242, ST144, ST1129, ST260, ST1743, ST3090, and ST1816 (each 2/66, 3.0%). The remaining 44 STs were represented by single isolates, including several novel or rarely reported sequence types. Three isolates (4.5%) could not be assigned to any known ST. O-antigen serotyping further demonstrated extensive diversity, with serotype O6 being the most prevalent (30/66, 45.5%), followed by O5 (10/66, 15.2%), O1 (9/66, 13.6%), and O7 (6/66, 9.1%). Based on colony morphology, 32 isolates were classified as mucoid and 34 as non-mucoid. Both mucoid and non-mucoid phenotypes were broadly distributed across phylogenetic clades II and III, with no apparent clustering pattern associated with the mucoid phenotype. Detailed phylogenetic annotations are provided in Table S1.

To further assess genetic relatedness and explore potential transmission events, we calculated pairwise SNP distances among all isolates based on whole-genome comparisons. The resulting distance matrix, visualized as a heatmap in Fig. S1, pairwise SNP distances ranged from 0 to 36,755, with a median of 16,023.5 SNPs. The majority of isolate pairs exhibited high SNP distances, consistent with the absence of dominant epidemic clones. Notably, only three isolate pairs showed very low SNP distances: NC523 and NC2526 (2 SNPs), NC373 and NC371 (0 SNPs), and NC531 and NC524 (4 SNPs), suggesting possible instances of hospital-associated transmission (Fig. S1). However, such genetically indistinguishable pairs were uncommon, indicating that *P. aeruginosa* infections in NCFB patients are largely sporadic and independently acquired rather than resulting from cross-transmission within the clinical setting.

### Adaptive genetic variations in *P. aeruginosa* isolates from NCFB patients

**Fig 2:**
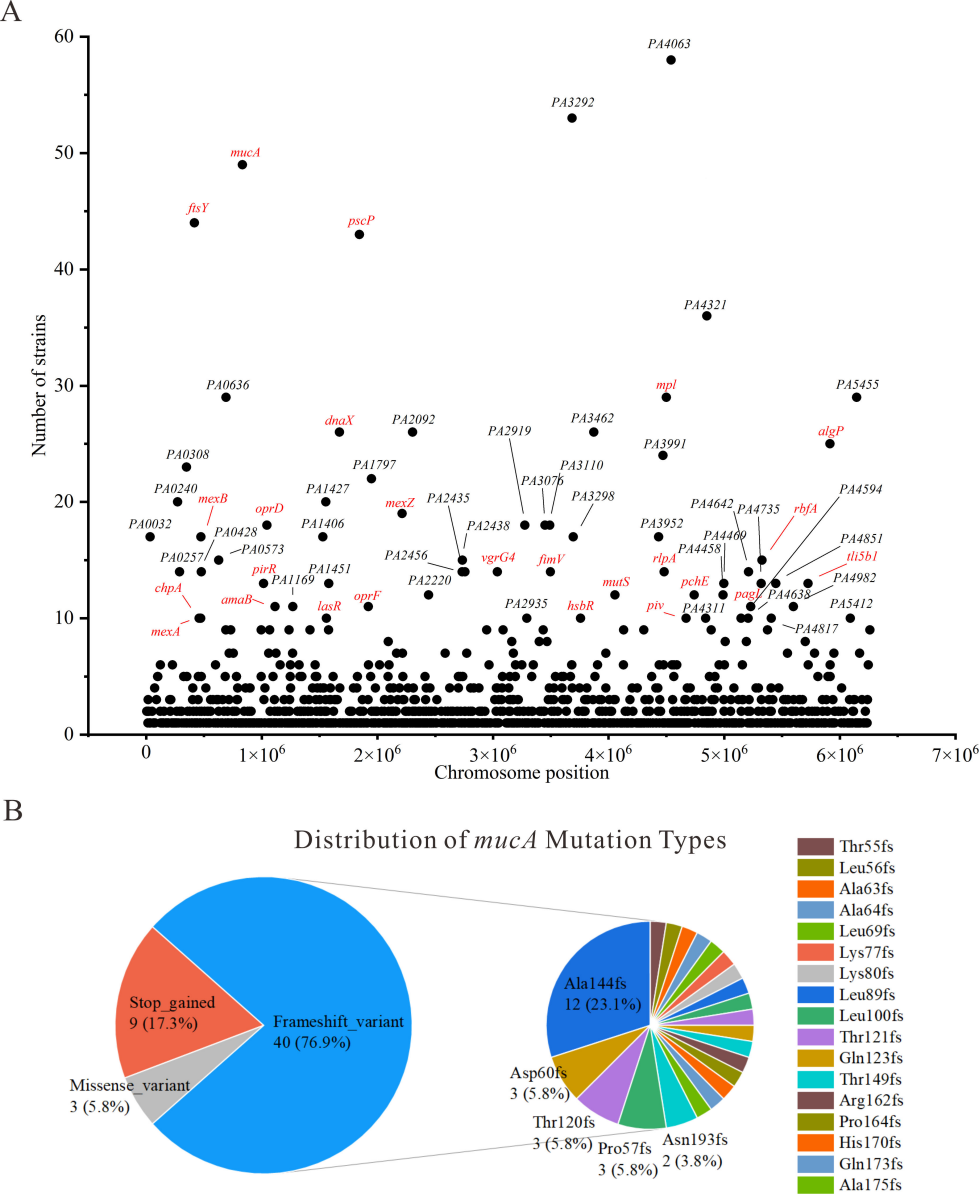
Adaptive LoF mutations and *mucA* allelic diversity of *P. aeruginosa* isolates from NCFB patients. (**A**) Frequency and chromosomal distribution of LoF mutations. Each dot represents a gene with a LoF mutation detected in 66 NCFB isolates. (**B**) Distribution of *mucA* mutation types among 52 *P*. *aeruginosa* isolates.

To elucidate the genetic strategies underlying chronic adaptation of *P. aeruginosa* in NCFB, we analyzed the prevalence and distribution of LoF mutations across 66 clinical isolates. The detailed frequencies and functional annotations of these genes are provided in Table S3. Notably, six genes, including *PA4063*, *PA3292*, *mucA* (a key regulator of mucoid conversion and chronic adaptation), *ftsY* (signal recognition particle receptor), *pscP* (translocation protein in type III secretion), and *PA4321*, exhibited LoF mutations in more than half of the 66 isolates (Fig. 2A). This finding indicates strong and consistent selective pressure favoring their inactivation within the NCFB airway environment. In addition, several other genes displayed a high prevalence of LoF mutations, each present in more than one quarter of the isolates. These genes include *PA0636*, *mpl*, *PA5455*, *dnaX*, *PA2092*, *PA3462*, *algP*, *PA3991*, *PA0308*, *PA1797*, *PA0240*, *PA1427*, *mexZ*, *oprD*, and *mexB*, among others. Comprehensive annotations of all LoF genes are provided in Table S4. These findings demonstrate that frequent LOF mutations in genes related to regulation, metabolism, and antibiotic resistance are a common adaptive feature among clinical NCFB isolates.

### Allelic diversity and mutation spectrum of *mucA* in *P. aeruginosa* isolates from NCFB patients

The *mucA* gene is a key regulator of alginate biosynthesis, and its mutation typically results in alginate overproduction and the emergence of the mucoid phenotype in *P. aeruginosa*. A total of 52 isolates harbored *mucA* mutations with substantial allelic diversity. Mutations in *mucA* were identified in 93.8% (30/32) of mucoid and 64.7% (22/34) of non-mucoid *P. aeruginosa* isolates. Fisher’s exact test revealed that *mucA* mutations were significantly more frequent among mucoid isolates compared with non-mucoid isolates (*P* = 0.02). As shown in Fig. 2B, frameshift variants accounted for the majority (40/52, 76.9%), followed by stop-gained mutations (9/52, 17.3%) and a smaller proportion of missense variants (3/52, 5.8%). Among the frameshift mutations, Ala144fs was the most prevalent, observed in 12 isolates (23.1%). Other frequent frameshift mutations included Pro57fs (3/52, 5.8%), Thr120fs (3/52, 5.8%), and Asp60fs (3/52, 5.8%), as well as Asn193fs (2/52, 3.8%). The remaining frameshift mutations (Thr55fs, Leu56fs, Ala63fs, Ala64fs, Leu69fs, Lys77fs, Lys80fs, Leu89fs, Leu100fs, Thr121fs, Gln123fs, Thr149fs, Arg162fs, Pro164fs, His170fs, Gln173fs, and Ala175fs) were each detected in a single isolate (1.9% each). Regarding stop-gained mutations, three distinct alleles were identified: Gln117* in five isolates, Gln118* in three isolates, and Gln128* in one isolate. For missense variants, Ala63Gly, Thr96Pro, and GluGln141GlyArg were each detected in one isolate.

### Genetic variation of virulence factors in *P. aeruginosa* isolates from NCFB

**Fig 3:**
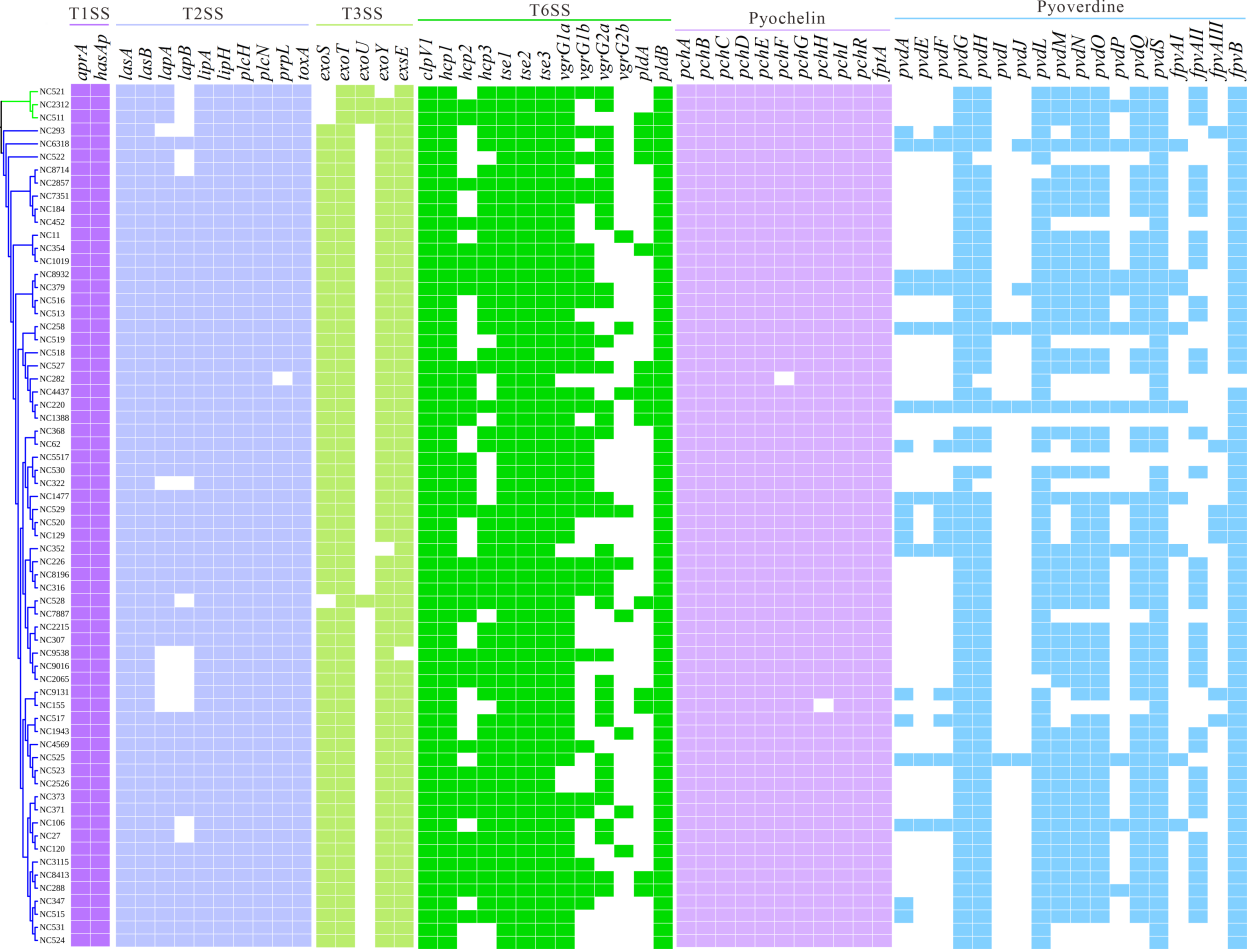
Heatmap of virulence gene presence/absence in 66 *P*. *aeruginosa* isolates from NCFB patients. Genes are grouped by functional category: T1SS, T2SS, T3SS, T6SS, pyochelin, alginate, and pyoverdine.

As shown in Fig. 3, all isolates carried the T1SS genes *aprA* and *hasAp*. Among the T2SS-related genes, *lasA*, *lasB*, *lipA*, *lipH*, *plcH*, *plcN*, and *toxA* were consistently present in all isolates, whereas *lapA*, *lapB*, and *prpL* were absent in some isolates. Regarding T3SS effectors, *exoT* (66/66, 100%), *exoY* (64/66, 97.0%), and *exoS* (62/66, 94.0%) were almost universally present, while *exoU* was detected in only four isolates (6.1%). Among T6SS-related genes, all isolates carried *clpV1, hcp1*, *tse1*, *tse2*, *tse3*, and *pldB*. In contrast, *hcp2* and *hcp3* were detected in 37 (56.1%) and 53 (80.3%) isolates, respectively. The *vgrG* gene family showed pronounced variability: *vgrG1a* was present in 62 isolates (93.9%), *vgrG1b* in 34 (51.5%), *vgrG2a* in 37 (56.1%), and *vgrG2b* in only 9 (13.6%) isolates. The phospholipase D gene *pldA* was detected in 15 isolates (22.7%).

Pyochelin biosynthesis and transport genes (*pchA-I*, *pchR*, and *fptA*) were almost universally present among the isolates. In contrast, genes related to pyoverdine synthesis and receptor systems exhibited substantial variation (Fig. 3). Based on the reference *fpvA* receptor sequences from *P. aeruginosa* PAO1 (*fpvAI*), ATCC 27853 (*fpvAII*), and ATCC BAA-2108 (*fpvAIII*), we determined the distribution of receptor types in our collection. Specifically, nine isolates carried *fpvAI* (13.6%), 38 possessed *fpvAII* (57.6%), and seven harbored *fpvAIII* (10.6%), while 12 isolates lacked any of these three *fpvA* variants.

The alginate biosynthesis genes were highly conserved among the isolates (Table S1). All strains carried *alg44*, *alg8*, *algA-L*, *algQ*, *algR*, and *algW* (66/66, 100%), while *algP* and *algU* were detected in 62 (93.9%) and 64 (97.0%) isolates, respectively. In contrast, considerable heterogeneity was observed in genes associated with bacterial motility. While *pilB* was present in all isolates (66/66, 100%) and *pilY1* in 59 (89.39%), *pilC* and *pilA* were found in 29 (43.9%) and 5 (7.6%) isolates, respectively. Among flagellar genes, *flgA* was detected in all isolates (66/66, 100%), but *fliC*, *fliD*, and *flgL* were only present in 29 (43.9%) each.

### Antibiotic resistance profiles and resistance determinants in *P. aeruginosa* from NCFB patients

**Fig 4:**
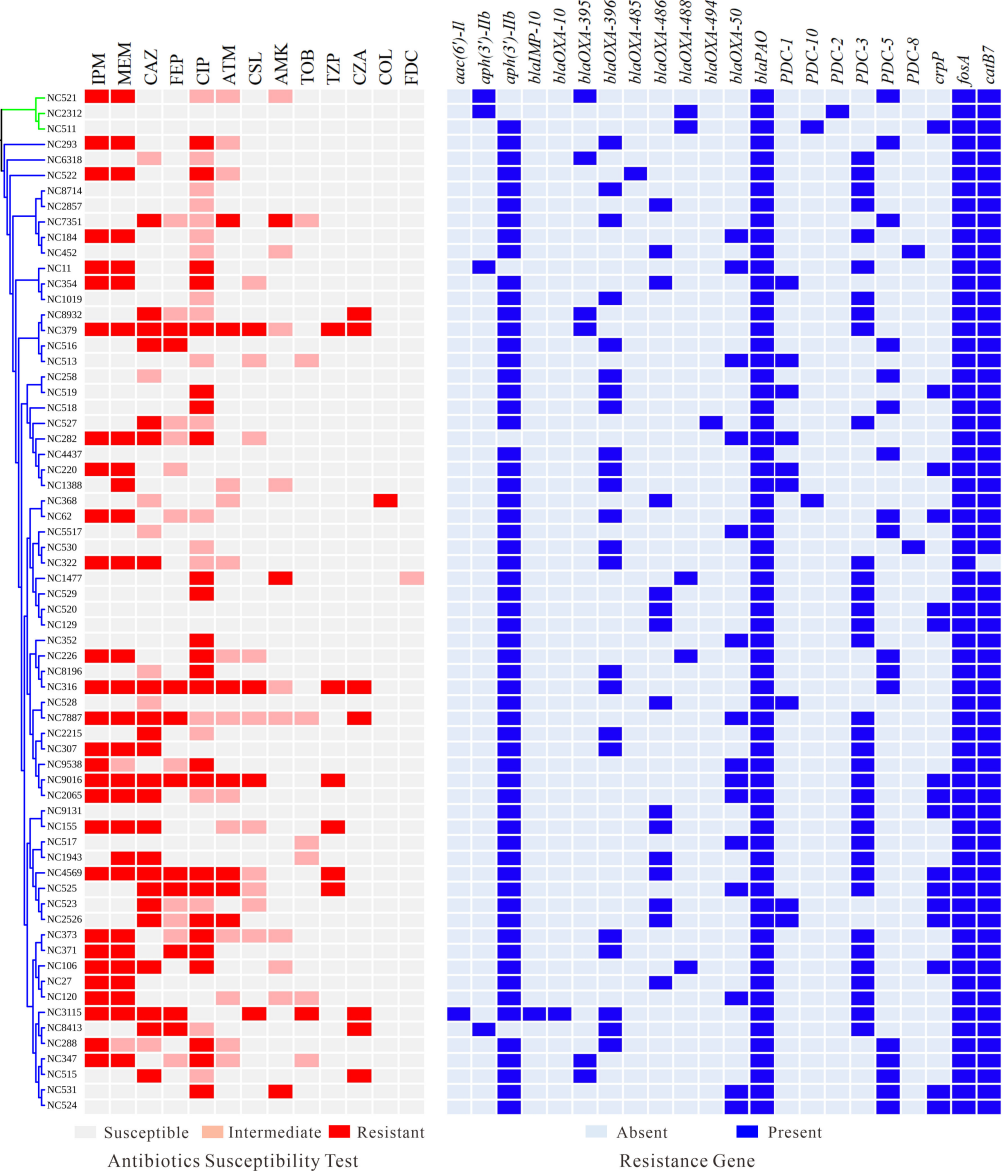
Heatmap displaying the antibiotic susceptibility profiles (left) and resistance gene profiles (right) among 66 *P*. *aeruginosa* isolates from NCFB patients.

Antimicrobial susceptibility testing of 66 *P*. *aeruginosa* isolates from NCFB patients demonstrated substantial variation in resistance phenotypes (Fig. 4). Overall, 45.5% (30/66) of isolates were resistant to at least one of the carbapenems, imipenem or meropenem. Resistance to ciprofloxacin was observed in 37.9% (25/66) of isolates, whereas 30.3% (20/66) exhibited intermediate susceptibility and 31.8% (21/66) remained susceptible. The proportions of isolates resistant to ceftazidime and cefepime were 34.8% and 15.2%, respectively. In contrast, resistance rates to other antimicrobial agents, including aztreonam, cefoperazone/sulbactam, amikacin, tobramycin, piperacillin/tazobactam, and ceftazidime-avibactam, ranged from 1.5% to 10.6%. Only one isolate was resistant to colistin, and one showed intermediate susceptibility to cefiderocol.

Genomic analysis revealed a diverse distribution of resistance determinants across the isolates (Fig. 4). Multiple resistance genes intrinsic to *P. aeruginosa* were identified across the isolates. The aminoglycoside resistance gene *aph(3')-IIb_2* was detected in 61/66 isolates (92.4%), *fosA* was present in all isolates, and *catB7* was found in 65 isolates (98.5%). Chromosomally encoded β-lactamase genes were also ubiquitous, with *bla*_OXA-50_ and its variants (*bla*_OXA-486_, *bla*_OXA-396_) detected in the majority of isolates (80.3%, 53/66). PDC alleles were observed, among which PDC-3 was present in 35 isolates (53.0%). In contrast, acquired resistance determinants were rare in these isolates. The aminoglycoside resistance genes *aac(6')-Il* and *aph(3')-IIb_1* were detected in one isolate (1.5%) and four isolates (6.1%), respectively. The acquired β-lactamase genes *bla*_OXA-10_ and *bla*_IMP-10_ were each present in a single isolate (1.5%). Although the quinolone resistance gene *crpP* was identified in 24.2% of isolates, its distribution did not fully account for the high frequency of CIP resistance. To further elucidate the genetic basis of fluoroquinolone resistance, we analyzed mutations in the quinolone resistance-determining regions (QRDRs) of *gyrA*, *gyrB*, *parC*, and *parE* in ciprofloxacin-resistant isolates (Table S5). QRDR mutations were detected in 72.0% (18/25) of resistant isolates, with the most common being *gyrA* T83I (52.0%), a well-characterized mutation known to confer high-level fluoroquinolone resistance. Additional mutations in *gyrA* (D87N/Y/H, S912_E913del, R237H) and frequent substitutions in *gyrB*, *parC*, and *parE* (S466F/Y, V646L, D533E) were also identified, frequently occurring in combination.

### Mucoid *P. aeruginosa* exhibits elevated cefiderocol MICs and enhanced biofilm formation, but reduced acute virulence

**Fig 5:**
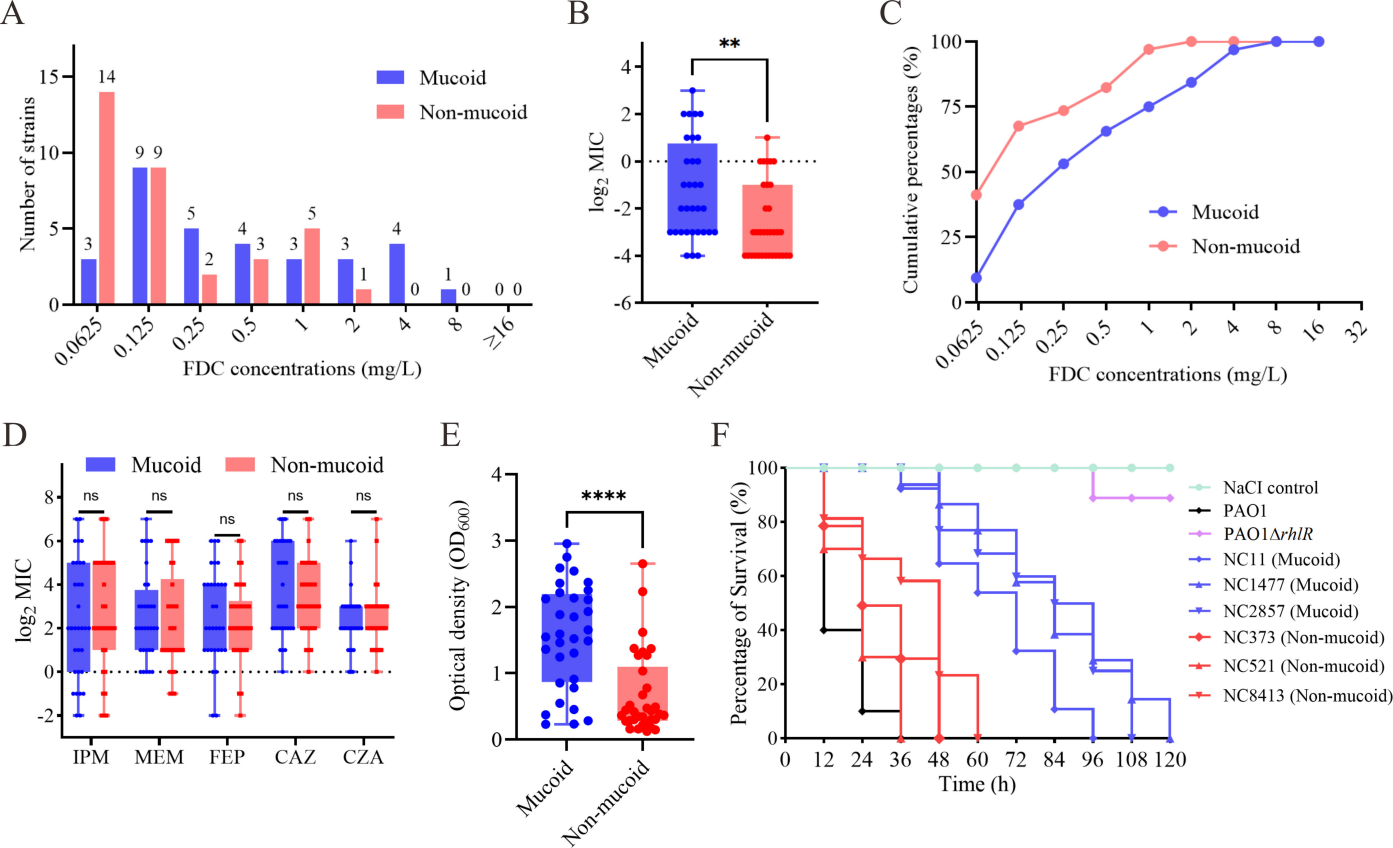
Phenotypic tests of the mucoid and non-mucoid isolates from NCFB patients. (**A**) Distribution of cefiderocol MICs among mucoid and non-mucoid *P. aeruginosa* clinical isolates. (**B**) Comparing cefiderocol MIC values between mucoid and non-mucoid isolates. Statistical analysis was performed using the unpaired Mann-Whitney test. (**C**) Cumulative frequency curves of cefiderocol MICs for mucoid and non-mucoid isolates. (**D**) Comparison of imipenem, meropenem, cefepime, ceftazidime, and ceftazidime-avibactam MICs between mucoid and non-mucoid isolates. Statistical analysis was performed using the unpaired Mann-Whitney test. (**E**) Quantification of biofilm formation by mucoid and non-mucoid isolates using the crystal violet assay. Statistical analysis was performed using the unpaired *t*-test. (**F**) Survival curves of *G. mellonella* larvae infected with representative mucoid and non-mucoid *P. aeruginosa* isolates. ** , **** , and ns indicate *P* < 0.01, *P* < 0.0001, and not significant (*P* > 0.05), respectively.

Although none of the 66 *P*. *aeruginosa* isolates met the CLSI breakpoint for cefiderocol resistance (MIC ≥ 16 mg/L), it is noteworthy that the five isolates exhibiting the highest cefiderocol MICs (4–8 mg/L) were all of the mucoid phenotype (Fig. 5A). This observation prompted us to investigate the association between the mucoid phenotype and reduced susceptibility to cefiderocol. The MIC_50_ and MIC_90_ values for cefiderocol in mucoid isolates were 0.25 and 4 mg/L, respectively, compared to 0.125 and 1 mg/L for non-mucoid isolates. Notably, the median cefiderocol MIC was significantly higher in the mucoid group than in the non-mucoid group (0.25 vs 0.125 mg/L; *P* = 0.0073; Fig. 5B). Cumulative frequency analysis further highlighted this reduced susceptibility, as mucoid isolates demonstrated a rightward shift in MIC distribution relative to non-mucoid isolates (Fig. 5C). To assess whether the mucoid phenotype was associated with reduced susceptibility to other β-lactams, MICs of imipenem, meropenem, cefepime, ceftazidime, and ceftazidime-avibactam were compared. As shown in Fig. 5D, no statistically significant differences in MIC values were observed between mucoid and non-mucoid isolates for any of these agents (*P* > 0.05 for all comparisons).

To compare the virulence of mucoid and non-mucoid *P. aeruginosa* isolates, quantitative biofilm assays and *G. mellonella* infection models were performed. As shown in Fig. 5E, mucoid isolates produced significantly greater biofilm biomass than non-mucoid isolates (*P* < 0.0001). Six clinical isolates were selected based on core-genome phylogenetic analysis, including three mucoid (NC11, NC1477, NC2857) and three non-mucoid (NC373, NC521, NC8413) strains from distinct phylogenetic branches (Fig. 1B). Genomic analysis of the six representative isolates revealed broadly similar virulence gene profiles, with all strains harboring key determinants, such as *lasA/B*, *toxA*, *plcH/N*, *pldB,* and T6SS associated genes (*clpV1*, *tse1-3*, *vgrG1a*). Minor variation was observed in type III secretion factors (*exoU*, *exoS*, *exoY*), T6SS components (*hcp2-3*, *vgrG1a*, *vgrG2a*, *vgrG2b*), *lapB,* and *pldA* (Fig. 3). *exoU* was detected exclusively in the non-mucoid strain NC521, while all other isolates lacked this gene. However, no consistent virulence gene differences were associated with the mucoid phenotype. In the *G. mellonella* infection model, non-mucoid isolates caused rapid larval death, with 100% mortality occurring within 36–60 h post-infection (Fig. 5F). In contrast, mucoid isolates showed significantly attenuated virulence, with complete mortality delayed to 96–120 h. Taken together, these findings indicate that mucoid *P. aeruginosa* isolates exhibit decreased susceptibility to cefiderocol, enhanced biofilm formation, and reduced acute virulence.

### Alginate overproduction contributes to elevated cefiderocol MICs in mucoid *P. aeruginosa*

**Fig 6:**
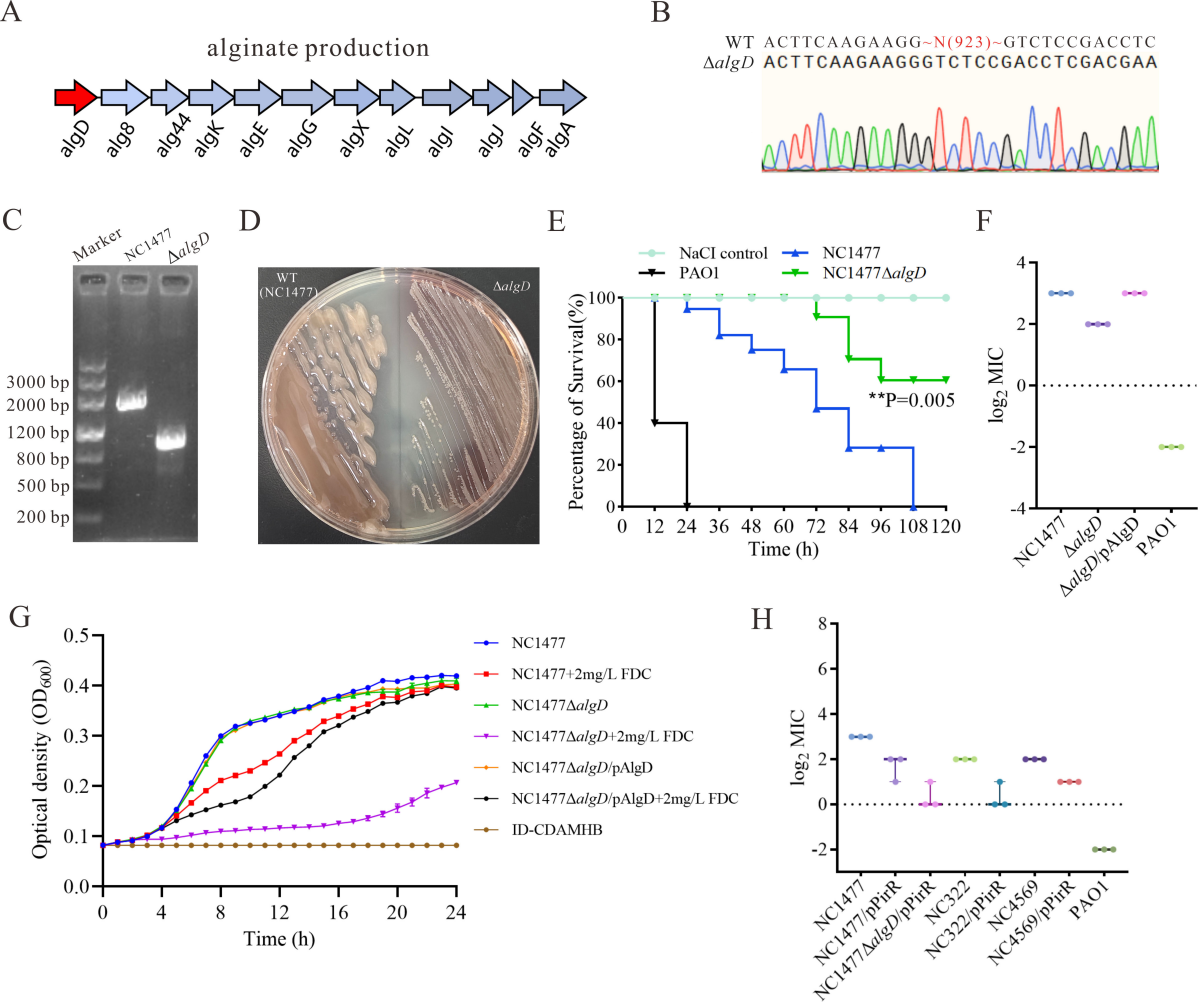
Synergistic effects of alginate overproduction and *pirR* mutation on cefiderocol susceptibility in mucoid *P. aeruginosa*. (**A**) Schematic representation of the alginate biosynthetic gene cluster in *P. aeruginosa*, with *algD* highlighted in red. (**B**) Sanger sequencing chromatograms confirming successful deletion of *algD* in the mucoid clinical isolate NC1477. (**C**) Agarose gel electrophoresis validating the knockout of *algD* in NC1477; DNA Marker: TIANGEN Marker DL2000 (MD103). (**D**) Representative colony morphologies of NC1477 wild-type (left) and *algD* deletion mutant (Δ*algD*, right) grown on LB agar. (**E**) Survival curves of *G. mellonella* larvae infected with NC1477 and Δ*algD*. *P* value was calculated using the log-rank (Mantel-Cox) test. (**F**) MICs of cefiderocol for wild-type and mutant strains, including complemented Δ*algD* (Δ*algD*/pAlgD) and PAO1, were each performed in biological triplicate. (**G**) Growth curves of NC1477, NC1477Δ*algD*, NC1477Δ*algD*/pAlgD, and PAO1 in the presence of sub-inhibitory concentrations of cefiderocol. Each curve represents the mean of three biological replicates, with error bars indicating the standard deviation. (**H**) MICs of cefiderocol for wild-type and mutant *P. aeruginosa* strains. MIC values represent the mean ± standard deviation from three independent experiments performed in biological triplicate.

The alginate biosynthetic operon in *P. aeruginosa* comprises 12 genes (*algD*, *alg8*, *alg44*, *algK*, *algE*, *algG*, *algX*, *algL*, *algI*, *algJ*, *algF*, and *algA*) that collectively govern the synthesis and export of alginate (Fig. 6A). This operon forms a contiguous gene cluster, with *algD*, encoding GDP-mannose dehydrogenase, serving as the rate-limiting step by catalyzing the conversion of GDP-mannose to GDP-mannuronic acid. To investigate whether alginate directly contributes to decreased cefiderocol susceptibility, we generated an *algD* deletion mutant in the mucoid clinical isolate NC1477, which displayed a relatively high cefiderocol MIC (8 mg/L). Successful deletion of *algD* was confirmed by Sanger sequencing and agarose gel electrophoresis (Fig. 6B and C), and phenotypic analysis revealed a clear transition from mucoid to non-mucoid colony morphology in the Δ*algD* mutant (Fig. 6D), indicating decreased alginate production. In the *G. mellonella* infection model, the *algD* deletion mutant exhibited significantly attenuated virulence compared to the NC1477 strain (*P* = 0.005; Fig. 6E). Antimicrobial susceptibility testing showed that the Δ*algD* mutant exhibited a twofold reduction in cefiderocol MIC (from 8 to 4 mg/L), whereas complementation with a plasmid-borne *algD* restored the high-level MIC to that of the parental strain (8 mg/L; Fig. 6F). To further assess the contribution of alginate biosynthesis to cefiderocol resistance, we compared the growth kinetics of the wild-type strain NC1477, the Δ*algD* mutant, and the complemented strain (Δ*algD*/pAlgD) in ID-CAMHB containing a sub-inhibitory concentration of cefiderocol (2 mg/L). As shown in Fig. 6G, the Δ*algD* mutant exhibited a marked reduction in growth rate in the presence of 2 mg/L cefiderocol, with OD_600_ values remaining significantly lower than those of the untreated control throughout the 24 h period. This growth impairment was particularly pronounced during the exponential phase, suggesting heightened sensitivity of the alginate-deficient strain to cefiderocol stress. In contrast, the wild-type strain and the complemented strain showed only transient growth inhibition during the logarithmic phase, indicating that alginate production mitigates the inhibitory effects of low-level cefiderocol exposure. However, deletion of *algD* results in only a modest reduction, suggesting that alginate is not the sole determinant of decreased cefiderocol susceptibility.

### Iron transport gene mutations and alginate overproduction synergistically reduce cefiderocol susceptibility in mucoid *P. aeruginosa*

Previous studies have indicated that the production of metallo-β-lactamases and mutations in iron transport genes can decrease the susceptibility of *P. aeruginosa* to cefiderocol (42). However, only one isolate was found to carry the *bla*_IMP-10_ metallo-β-lactamase (Fig. 4). To explore whether mutations in iron transport systems contribute to reduced cefiderocol susceptibility, we performed SNP analysis of iron transport-related genes in five clinical isolates with high cefiderocol MICs (≥4 mg/L; Fig. 5A). All five isolates harbored non-synonymous or frameshift mutations in at least one iron acquisition-associated gene, with a diverse constellation of mutational profiles observed (Table 1). Frequent alterations were observed in *fptAI*, *chtA*, and *pfeA*, with each strain displaying a unique combination of mutations (Table 1). Notably, three isolates (NC322, NC1477, NC4569) shared a frequent frameshift mutation in *pirR* (Gly132fs), which may represent a convergent mechanism contributing to impaired iron regulation and reduced cefiderocol susceptibility. To clarify the role of *pirR* in cefiderocol resistance, we constructed *pirR* complementation plasmids and introduced them into the three clinical isolates carrying the *pirR* (Gly132fs) mutation (NC322, NC1477, NC4569). Notably, complementation of *pirR* resulted in a significant reduction in cefiderocol MICs for all three strains (Fig. 6H). Specifically, in NC1477, the MIC decreased from 8 mg/L to 2–4 mg/L, while in NC322 and NC4569, MICs declined from 4 mg/L to 1–2 mg/L. To investigate the relationship between alginate production and iron transport gene-mediated resistance, we also complemented *pirR* in the alginate-deficient background (NC1477Δ*algD*). Notably, the MIC in NC1477Δ*algD*/pPirR decreased even further, reaching 1–2 mg/L, which is close to the level observed in PAO1 (0.25 mg/L). This suggests that decreased sensitivity to cefiderocol in mucoid *P. aeruginosa* results from the combined effects of alginate overproduction and mutations of iron transport-related genes.

**TABLE 1:** Mutations in iron transport-associated genes identified in five clinical isolates with elevated cefiderocol MICs (≥4 mg/L)^*a*^

Strain	FDC MIC (mg/L)	Identified mutations in iron transport genes associated with cefiderocol resistance									
		*chtA*	*cirA*	*fecI*	*femA*	*fptA*	*fpvAI*	*fpvB*	*pirA*	*pirR*	*pfeA*
NC322	4	–	–	–	–	Pro407Arg; Pro561Ser	–	–	–	Gly132fs	Ala5Thr
NC516	4	Asp216Asn	–	–	–	Val373Leu	–	–	–	Gly135Asp; Leu168Gln	–
NC1477	8	–	–	Glu94Val	Pro500Leu	–	–	Val726Gly	–	Gly132fs	–
NC2526	4	Ser87Pro; Pro280Thr	Thr171Met	–	Pro373His	–	–	–	–	–	His456Arg
NC4569	4	Ser214Asn; Glu308Asp; Val612Leu	Asp247Gly	–	–	Gln115Arg	Val522fs	–	Val65fs	Gly132fs	Glu396Lys

^*a*^ Mutations were detected using PAO1 as the reference genome. To identify mutations specifically associated with cefiderocol resistance, isolates with low MICs (≤0.25 mg/L) were also analyzed, and mutations present in both resistant and susceptible isolates were excluded from further analysis. – indicates that no mutation is present.

## DISCUSSION

This study provides a comprehensive genomic characterization and adaptive landscape of *P. aeruginosa* isolated from patients with NCFB. Our results demonstrate significant genetic diversity among *P. aeruginosa* isolates from NCFB patients, with high SNP distances indicating that closely related lineages are rarely shared and that most infections originate from independent environmental acquisition rather than patient-to-patient transmission. This finding contrasts with the emergence and spread of dominant clonal lineages among CF populations, such as the M3L7 subtype of the AUST-02 shared strain in Australia and the Liverpool epidemic strain in the UK, both of which have been frequently identified among patients attending the same CF centers (43, 44). The extensive genetic diversity observed among isolates suggests that traditional infection control strategies, particularly patient isolation protocols, likely offer minimal benefit in preventing *P. aeruginosa* transmission within the NCFB patient population.

Our study also reveals that the adaptive landscape of *P. aeruginosa* in NCFB is shaped by strong selection for loss-of-function mutations, particularly in *mucA*, which drives the emergence of the mucoid phenotype. This observation is consistent with the previous findings in CF cohorts (45, 46), demonstrating that *P. aeruginosa* encounters comparable selective pressures during chronic airway infection across different underlying pulmonary conditions. Mutations in *mucA*, a hallmark of chronic infection adaptation, promote bacterial persistence, exacerbate airway inflammation, and are associated with worse clinical prognosis (21, 47, 48). The notable diversity of *mucA* mutations observed in our study, especially the dominance of frameshift mutations, such as Ala144fs, indicates that *P. aeruginosa* can achieve this adaptive phenotype through multiple genetic routes, which exemplifies convergent evolution. Prolonged exposure to diverse antibiotics for managing *P. aeruginosa* infections in bronchiectasis can shape the resistome and promote multidrug resistance (49). Our findings indicate that recurrent inactivation of *mexZ* and *oprD* in *P. aeruginosa* represents direct adaptation to antibiotic pressure, with *mexZ* inactivation leading to upregulation of multidrug efflux systems and *oprD* inactivation reducing antibiotic import through the outer membrane (50, 51). In addition, we identified loss-of-function mutations in genes involved in secretion systems, transcriptional regulation, and metabolism. These findings are in accordance with the “adaptive trade-off” hypothesis proposed by Ferenci et al. (52), which suggests that *P. aeruginosa* selectively reduces certain functions to optimize adaptation to specific environments.

Moreover, we observed that motility-associated genes exhibited substantial heterogeneity, with critical components such as *pilA* and flagellar genes (*fliC*, *fliD*, *flgL*) frequently undetectable in these isolates. This apparent absence may result from sequence divergence relative to the PAO1 reference genome, rather than true genomic deletion. A mutually exclusive distribution of *exoS* (93.94%) and *exoU* (6.06%) was observed, consistent with the established paradigm that these effectors are mutually exclusive and represent distinct virulence strategies (53). The predominance of *exoS*-positive strains in our NCFB cohort reflects adaptation to chronic colonization rather than acute cytotoxic infection (54). Antimicrobial susceptibility testing revealed concerning resistance profiles, with notably high resistance rates to ciprofloxacin and imipenem. The molecular basis of carbapenem resistance was complex, as only 1.5% of isolates carried carbapenemase genes, indicating that non-enzymatic mechanisms, including *oprD* inactivation and efflux upregulation, are the major contributors.

In our collection, mucoid and non-mucoid isolates were nearly equally distributed, indicating that both phenotypes can successfully colonize the airways of NCFB patients. Notably, our results show that mucoid isolates possess greater biofilm-forming capacity and reduced acute virulence, which reflects an evolutionary strategy favoring persistent infection over acute disease. From an ecological perspective, this adaptation is advantageous, as decreased acute virulence may help the bacteria evade strong host inflammatory responses, while enhanced biofilm formation promotes resistance to antimicrobial agents and host immune clearance (55, 56). The significance of this phenotypic switch to chronic infection was demonstrated in previous research, where mucoid *P. aeruginosa* isolates from persistent infections displayed reduced clearance rates in murine lung models (57). Consistent with this, our *G. mellonella* model results further indicate that although mucoid isolates form robust biofilms, they exhibit markedly lower acute virulence, which can be attributed to the regulatory interplay between the *mucA*/*kinB* pathway and the sigma factors AlgU and AlgR (58, 59). Loss of function of *mucA* leads to constitutive activation of AlgU, which promotes alginate biosynthesis while repressing the expression of acute-phase virulence factors, including the type III secretion system and exotoxin A (59). Notably, deletion of *algD* in a mucoid clinical isolate reduced virulence, indicating that alginate overproduction positively contributes to the pathogenicity of mucoid *P. aeruginosa*. These findings have important clinical implications for NCFB management. The presence of mucoid *P. aeruginosa* may indicate a higher risk of persistent, difficult-to-eradicate infection and is often associated with declining lung function in NCFB patients (19). Effective management may therefore require optimized antibiotic strategies and the adjunctive use of anti-biofilm or mucolytic agents.

Our study reveals an important observation that mucoid *P. aeruginosa* isolates demonstrate reduced susceptibility to cefiderocol, a novel siderophore cephalosporin designed to circumvent classical resistance mechanisms. Although none of the isolates reached the CLSI-defined resistance threshold (MIC ≥ 16 mg/L), mucoid isolates demonstrated significantly elevated MICs compared to non-mucoid isolates. In contrast, no significant differences were observed between mucoid and non-mucoid isolates in their susceptibility to imipenem, meropenem, cefepime, ceftazidime, or ceftazidime-avibactam. This phenotype-specific elevation in cefiderocol MICs is therefore unlikely to reflect a generalized β-lactam resistance trend. Instead, this finding may be attributed to cefiderocol’s distinctive “Trojan horse” mechanism (22), in which siderophore-mediated active transport facilitates drug entry into bacterial cells. In mucoid strains, the extracellular alginate matrix may partially impede this process and thereby increase MIC values. This observation is particularly noteworthy given that cefiderocol has only recently been introduced into clinical practice and has not yet been used in China. Our mechanistic investigations provide evidence that reduced cefiderocol susceptibility arises from a synergistic interplay between alginate overproduction and mutations in iron transport genes. The alginate-deficient mutant (Δ*algD*) exhibited a modest twofold reduction in cefiderocol MIC, suggesting that alginate contributes to reduced susceptibility but is not the sole determinant. This aligns with the findings of Hatch et al. (60), who demonstrated that alginate primarily functions as a diffusional barrier that limits antibiotic penetration. Importantly, clinical isolates with higher cefiderocol MICs commonly carried non-synonymous or frameshift mutations in multiple iron transport genes, notably a frequent frameshift mutation in *pirR* (Gly132fs), which has been associated with decreased cefiderocol susceptibility (61). Complementation of *pirR* experiments definitively established that restoration of functional *pirR* substantially enhanced cefiderocol susceptibility, with the most pronounced effect observed in the alginate-deficient background (NC1477Δ*algD*/pPirR). This finding indicates a mechanistic synergy wherein alginate impedes extracellular drug diffusion, while iron transport alterations diminish cellular uptake of this siderophore antibiotic. While cefiderocol remains active against most NCFB-derived *P. aeruginosa* isolates, those displaying the mucoid phenotype or harboring mutations in iron transport genes may exhibit reduced susceptibility, and higher dosing or combination therapy should be considered for these cases.

Several limitations should be acknowledged. Our single-center sample may not capture the full global diversity of NCFB-derived *P. aeruginosa*, and the lack of longitudinal sampling limits insights into the temporal evolution of adaptive changes. Future multicenter and longitudinal studies are needed to validate and expand upon these findings. In addition, future homoplasy analyses may provide further insights into convergent evolutionary patterns among NCFB-derived *P. aeruginosa*.

In summary, this study demonstrates that NCFB-derived *P. aeruginosa* undergoes extensive, parallel adaptive evolution, marked by diverse *mucA* mutations and a shift toward mucoid, biofilm-forming phenotypes, as well as the emergence of novel, non-enzymatic cefiderocol resistance mechanisms. These findings enhance our understanding of bacterial adaptation in chronic respiratory infections and identify potential targets for personalized therapy in NCFB patients with *P. aeruginosa*.

## Data Availability

The genome sequence data for 66 *Pseudomonas aeruginosa* isolates in this study have been submitted to the GenBank database at the National Center for Biotechnology Information (NCBI) (https://www.ncbi.nlm.nih.gov/nuccore/) under accession number PRJNA1258701.

## SUPPLEMENTAL MATERIAL

Figure S1 msystems.01514-25-s0001.docx Pairwise SNP distance matrix of all *P. aeruginosa* isolates from NCFB patients.

Table S1 msystems.01514-25-s0002.xlsx Molecular characteristics, antimicrobial resistance genes, and virulence gene profiles of 66 *P. aeruginosa* isolates from NCFB patients.

Table S2 msystems.01514-25-s0003.xlsx Primers used in this study.

Table S3 msystems.01514-25-s0004.xlsx Details of all loss-of-function mutation sites identified in 66 *P. aeruginosa* isolates from NCFB patients.

Table S4 msystems.01514-25-s0005.xlsx Annotations and mutation frequencies for all genes with LoF mutations in 66 *P. aeruginosa* isolates from NCFB patients.

Table S5 msystems.01514-25-s0006.xlsx Mutations detected in QRDRs of 25 ciprofloxacin-resistant *P. aeruginosa* isolates from NCFB patients.

ASM does not own the copyrights to Supplemental Material that may be linked to, or accessed through, an article. The authors have granted ASM a non-exclusive, world-wide license to publish the Supplemental Material files. Please contact the corresponding author directly for reuse.
