# Supplementary material for: Genomic diversity and adaptive resistance mechanisms in Pseudomonas aeruginosa from bronchiectasis
Source: mSystems. 2025 Dec 5;11(1):e01514-25. doi: 10.1128/msystems.01514-25 (PMC12817961; doi:10.1128/msystems.01514-25)
Supplement: Figure S1 — Pairwise SNP distance matrix of all P. aeruginosa isolates from NCFB patients. [file msystems.01514-25-s0001.docx]

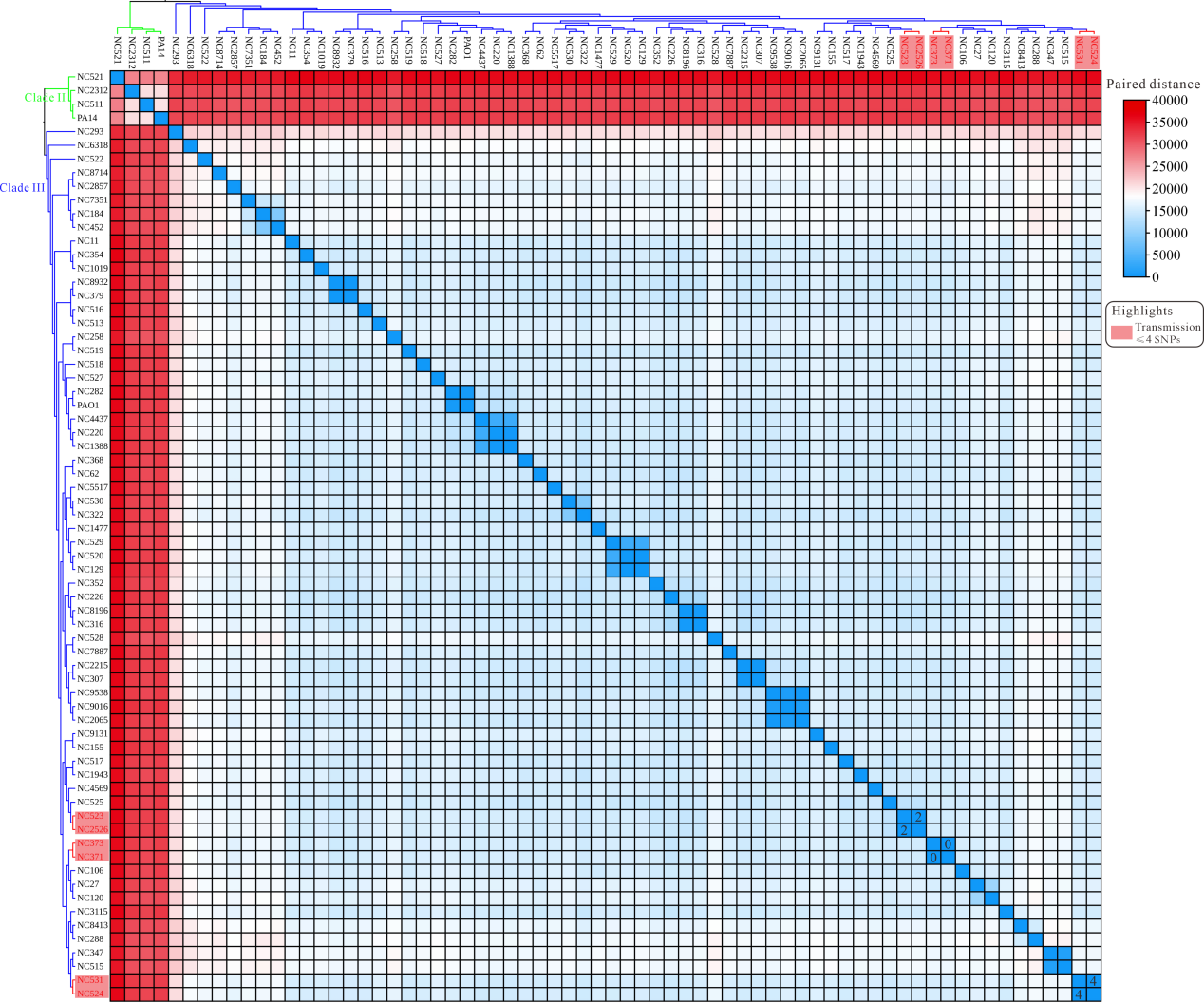


**Figure S1.** Pairwise SNP distance matrix of all *P. aeruginosa* Isolates from NCFB Patients. PA7 was excluded due to extremely high SNP distances to all other isolates.
